# Parenting Stress in Mothers of Children and Adolescents with Down Syndrome

**DOI:** 10.3390/jcm11051188

**Published:** 2022-02-23

**Authors:** Elisa Fucà, Floriana Costanzo, Luciana Ursumando, Stefano Vicari

**Affiliations:** 1 Child and Adolescent Neuropsychiatry Unit, Department of Neuroscience, Bambino Gesù Children’s Hospital, IRCCS, 00146 Rome, Italy; elisa.fuca@opbg.net (E.F.); luciana.ursumando@opbg.net (L.U.); stefano.vicari@opbg.net (S.V.); 2 Department of Life Sciences and Public Health, Catholic University, 00168 Rome, Italy; 3 Casa San Giuseppe, Centro di Riabilitazione Opera Don Guanella, 00165 Rome, Italy

**Keywords:** trisomy 21, Parenting Stress Index, socio-demographics, externalizing behaviors, internalizing behaviors, maternal employment

## Abstract

Parenting stress has deleterious effects on parents, children, and overall family functioning. Parents of children with intellectual disability, including Down Syndrome (DS), show higher levels of parenting stress than parents of typically developing children. This research aimed to (i) evaluate parenting stress levels in a group of mothers of youths with DS using a parent-report questionnaire, (ii) identify children’s individual and clinical features associated with maternal stress, and (iii) identify specific situational life/demographics factors related to maternal stress. Seventy-eight youths with DS underwent a neuropsychological evaluation, whereas mothers completed questionnaires for the assessment of parenting stress and of the child’s emotional and behavioral problems. We found that Parent–Child Difficult Interaction was the domain with the highest percentage of clinical scores (39.7%). Both internalizing and externalizing problems correlated with maternal stress, as well as autistic symptoms. The levels of maternal stress were not associated with any socio-demographic variable. After controlling for child-related correlates of maternal stress and for mothers’ age and education level, unemployed mothers exhibited higher levels of parental distress than employed mothers. The present study highlights that unemployment is related with parenting stress and potentially amenable to policy interventions supporting parents in combining work and family care.

## 1. Introduction

Parenting stress has been defined as “the disparity between the perceived demands of parenting and the resources parents have available to meet those demands” [1]. According to the model developed by Abidin in 1995 [2], parenting stress comprises three major domains: parent features, child features, and situational life/demographics. The three domains are reflected in the instrument Parenting Stress Index (PSI), designed to evaluate the different sources and facets of parental stress [2,3].

Parenting stress has deleterious effects on parents, children and overall family functioning. On one hand, stress can negatively influence parents’ mental health and wellbeing; several studies have reported an association between chronic stress and poor psychological and physical health in caregivers, as well as reduced satisfaction with life [4,5,6,7]. On the other hand, parental psychological stress can also affect children’s wellbeing, as well as psychological and developmental outcomes [8,9,10,11,12]. Several authors, indeed, reported associations between parenting stress and less stimulating interactions with child, as well as increased risk for child maltreatment and adverse childhood experiences [13,14,15,16,17,18,19,20]. Moreover, parenting stress occurring in the child’s early life seems to predict internalizing and externalizing problems [21]; for instance, Buodo and colleagues [22] reported a significant relationship between parenting stress and the children’s externalizing behavior in a group of 61 mothers with children aged 9–12 years.

The literature has clearly demonstrated that parents of children with intellectual disability (ID), as well as other neurodevelopmental disorders (NDDs), experience significantly higher levels of parenting stress than parents of typically developing (TD) children [23,24,25,26,27,28,29,30,31].

However, comparative studies have suggested that the causes of parental stress could be pathology-specific. For instance, in a study comparing stress in parents of children with Down Syndrome (DS), autism spectrum disorder (ASD), and Williams syndrome (WS), Ashworth and colleagues [32] reported that, despite comparable levels of parental stress, parents of children with ASD were significantly more stressed by contextual factors such as the difficulties to access a professional. Conversely, stress levels were more influenced by the severity of the symptoms in parents of children with DS or WS than those of children with ASD. A more recent study using a cross-disability approach found that emotion expression (in particular, the expression of high criticism) was differently associated with parenting stress in parents of children with DS compared with parents of children with other forms of disability [33].

Evidence in the literature suggests the existence of condition-specific peculiarities concerning not only the factors associated with maternal stress, but also the trajectories of maternal stress over time. It has been reported, indeed, that mothers of children with DS aged 3 years exhibit lower stress than mothers of children with ASD, cerebral palsy, and undifferentiated developmental delay [34]. However, the levels of maternal stress in mothers of children with DS tend to increase at ages 4 and 5 [34]. Similarly, Most and colleagues [35] investigated the levels of stress in a group of mothers of children with DS at 12–15, 30, and 45 months of age. The authors reported that maternal stress increased over these three time-points; of note, such an increase was not reported among the mixed-etiology comparison group. However, research comparing the parenting stress levels in caregivers of children *versus* adults with DS reported higher levels of parenting stress in caregivers of children compared with caregivers of adults [36].

In sum, it emerges that the proper identification of the specific factors affecting parental stress in DS could be the first necessary step for the setting up of adequate interventions. Parents of children with DS experience significantly lower levels of parenting stress than parents of children with other NDDs do, such as ASD [33,37,38,39,40,41], Prader–Willi syndrome [42], 22q11.2 deletion syndrome [39] and other forms of ID [26,43]. This phenomenon has been described in terms of a *Down Syndrome advantage* [44], according to which families of children with DS experience greater wellbeing than families of children with other kinds of ID. The *Down Syndrome advantage* has been traditionally explained with reference to some specific features of children with DS, which are usually described as being sociable, cheerful, and affectionate [45,46]. These features may foster positive interactions and lead parents to have better perceptions of their child.

Nevertheless, some factors associated with higher levels of parenting stress in DS have been identified. In particular, high levels of parental stress in DS have been associated with some child features, such as child’s age [26,34,35,42], low social desirability [34], and child behavioral problems [47], particularly externalizing behaviors and challenging behaviors, such as irritability and hyperactivity [43,48]. Research on the association between children’s internalizing behaviors and parental stress in DS provided inconsistent results, with some studies reporting weak relationships [48], and others reporting associations between the degree of the child’s internalizing behaviors and parent-related stress [26]. However, some research reported a small contribution of the child’s behavioral problems per se to variance of parenting stress [49,50]. Thus, the role of children’s behavioral problems, particularly the possible differential effect of internalizing *versus* externalizing behaviors on maternal stress, remains unclear in DS.

On the other hand, less information is available about the role of other child’s features, such as sex, IQ, and adaptive abilities, with some evidence for a lack of association between parental stress and child’s sex [26] and for a relationship with the level of child disability [51]. Moreover, since parents of children with ASD have been consistently reported as highly stressed compared with parents of children with DS [37,41], it would be interesting to investigate whether the presence of ASD-related symptoms in the absence of a formal diagnosis of ASD in children with DS could affect the levels of parental stress.

Beyond the child’s features, parental features, such as parenting style and parental coping strategies, should be considered important factors linked with parenting stress in DS. For instance, some studies reported that emotion-oriented coping (i.e., the tendency of responding to stress with self-oriented emotional reactions, such as becoming upset) and avoidance coping styles are predictors for parental stress among parents of children with DS [38,52]. Moreover, other studies found that parental coping style significantly correlates with parenting stress in mothers and caregivers of children with DS [36,49]. On the other hand, it has been reported that parenting stress could act as a mediator of differences in parenting styles between mothers of children with DS and typically developing children [53].

Lastly, the role of socioeconomic status (SES) should not be underestimated when considering the factors associated with parental stress in DS. Indeed, families of youths with ID spend large amounts of time on providing care [54]. In order to provide this care, many parents need to adjust their work and family balance. Consistently, previous research documented that parents of children with disabilities are more likely to not be in paid employment or to have reduced working hours, because of their requirements to provide care for their child [55,56,57,58,59]. Specifically, mothers of children with DS tend to be more often employed part-time than mothers of TD children [60]. Therefore, families of children with ID are at considerably increased risk of exposure to adverse socioeconomic conditions [61,62,63], with a significant impact on parental happiness, self-esteem, and self-efficacy [64]. Of note, SES has been identified as one of the crucial factors influencing parenting stress in NDDs. Bonifacci and colleagues [65] reported that lower SES and number of family members predicted parenting stress in a sample of 130 parents of children with attention deficit/hyperactivity disorder (ADHD) and dyslexia. Similarly, family income negatively correlated with parenting stress and parental stress in parents of children with ID [66], epilepsy [67], and Tourette syndrome [68]. In contrast with the literature on other NDDs, previous research on DS did not report an association between family income and maternal stress [49].

On the other hand, findings on the effect of parents’ educational level on parenting stress in NDDs are not consistent. Whilst some authors reported that educational level negatively predicts parenting stress in parents of children with NDDs [3,69], other studies failed to detect such association [70,71]. Given the greater wellbeing consistently reported in parents of children with DS in comparison to other forms of ID, Stoneman [72] investigated the extent to which differences in family income and child temperamental difficulty can explain why parents of children with DS experience higher levels of wellness. The author reported that family income was more important than etiology in predicting parental wellbeing. However, poor knowledge is available about the association between the unemployment condition *per se* and maternal stress in DS.

This research had the general objective of evaluating the relationship between maternal stress levels in youths with DS and several factors related to child characteristics and situational life/demographic conditions, studied simultaneously in the same sample. Child characteristics included individual features (sex, age, IQ, adaptive level) and emotional and behavioral symptoms assessed by parent-report questionnaires (internalizing, externalizing, and social communication problems). Situational life/demographic factors included family SES, maternal age and years of education, and maternal employment. Some of these factors, such as social communication problems and maternal employment status, were not studied previously in relation with maternal stress in DS. Data were retrospectively collected from previous clinical evaluations.

Specific aims of the study were (i) evaluating parenting stress levels in a group of mothers of youths with DS through a parent-report questionnaire, (ii) identifying children’s individual and clinical features associated with maternal stress, and (iii) identifying specific situational life/demographics factors related with maternal stress. We hypothesized that (i) the levels of total stress in mothers of youths with DS would be low, consistently with what has been previously reported in the literature, (ii) maternal stress would increase as the child’s age increased and would be significantly associated with social communication difficulties and with externalizing but not internalizing behaviors, as assessed by parent-report instruments, and (iii) SES, but not maternal education, would be negatively correlated with maternal stress.

## 2. Materials and Methods

### 2.1. Participants

Seventy-eight mothers of youths with DS ranging in age from 25.27 to 58 years (mean 44.59 ± 6.98 years) were included in the study. Thirty-one women out of 78 (40%) were not employed at the time of the evaluation; the mean level of education was 13.33 ± 3.84 years of schooling. The patients included in the study (54 boys, 24 girls) ranged in age from 3.5 to 18 years, with a mean age of 9.64 ± 3.95 years; the mean IQ was 55.55 ± 9.13. Selection criteria included, in addition to the diagnosis of DS based on the analysis of the karyotype, an age ranging between 3 and 18 years. Exclusion criteria were as follows: the ascertained presence or the clinical suspect of neurological conditions; the presence of neurodevelopmental or psychiatric comorbidities (e.g., ASD, ADHD); language barrier hampering questionnaire compilation by parents. All participants underwent a child psychiatric and neuropsychological examination conducted by experienced developmental neuropsychiatrists and neuropsychologists. The absence of neurological disorders was determined through a neurological examination and collection of clinical history, whereas the absence of neuropsychiatric comorbid conditions was determined via direct observation and administration of clinical interviews to caregivers, such as the Schedule for Affective Disorders and Schizophrenia for School-Age Children—Present and Lifetime Version [73].

### 2.2. Procedure

This is a cross-sectional, observational study; data were retrospectively collected from a file review of patients with DS referred for a clinical evaluation at the Child and Adolescent Neuropsychiatry Unit of the Bambino Gesù Children’s Hospital in Rome between February 2021 and October 2021. The relationship between maternal stress levels in children and adolescents with DS and several variables related to child characteristics and situational life/demographic conditions was analyzed. Children’s characteristics included sex, age, nonverbal IQ, adaptive level, and internalizing, externalizing, and social communication problems. Situational life/demographic conditions included family SES, maternal age and years of education, and maternal employment. The clinical evaluation of children and adolescents with DS consisted of a neuropsychiatric, neuropsychological, and psychopathological/behavioral assessment performed by a team consisting of a child neuropsychiatrist and clinical psychologists and neuropsychologists with clinical expertise on DS. The clinical evaluation also included the administration of parent-report measures.

Data were collected from the hospital records and clinic charts, and the de-identified data were entered into an Excel spreadsheet. The majority of the data collected were quantitative (e.g., scores obtained at standardized tests and parent-report questionnaires) and not subject to interpretation. For the current project, all subjects meeting specified criteria as described above were selected from a database. The study was conducted according to the guidelines of the Declaration of Helsinki. All data were de-identified, and patients’ confidentiality was protected; for this type of study, informed consent was not required.

### 2.3. Measures

#### 2.3.1. Maternal Stress

The Parenting Stress Index—Short Form (PSI-SF) [2,74,75] is an easy-to-administer tool to measure maternal stress. It consists of 36 questions, and each item is rated on a five-point Likert scale from (1) strongly disagree to (5) strongly agree. The PSI-SF captures three domains—parental distress, parent–child dysfunctional interaction, and difficult child. The Parental Distress (PD) subscale evaluates the “distress that a parent is experiencing in their role as a parent” [74]. The Difficult Child (DC) subscale assesses parenting challenges related to a child’s self-regulation or behavioral difficulties. The Parent–Child Dysfunctional Interaction (P-CDI) subscale is designed to assess unsatisfactory parent–child interactions. The sum of all questions results in the total stress score.

#### 2.3.2. Cognitive Measures

Cognitive development was tested by the Leiter-3 [76], which provides a nonverbal measure of intelligence and assesses the ability to reason by analogy, matching, and perceptual reasoning, irrespective of language and formal schooling for individuals aged 3–70. The Griffiths III [77] was administered only in few cases (eight participants) when the child failed to complete the Leiter-3 because of their reduced attentional resources.

#### 2.3.3. Adaptive Abilities

To assess the presence of impairments in adaptive behaviors necessary for socialization, communication, and daily functioning, we used the Adaptive Behavior Assessment System II (ABAS-II) [78], a parent/primary caregiver questionnaire. ABAS-II consists of 11 skill areas organized into three general domains: conceptual, practical, and social. The composite and domain scores are standard scores with a norm-referenced mean of 100 and standard deviation of 15.

#### 2.3.4. Emotional and Behavioral Problems and Social Communication Abilities

The Child Behavior Checklist (CBCL) [79] was used to assess emotional and behavioral problems. For preschool children, we used the CBCL for ages 1.5 to 5, which consists of 100 problem items. There are seven syndrome scales: emotionally reactive, anxious/depressed, somatic complaints, withdrawn, sleep problems, attention problems, and aggressive behavior. The summary profile contains the internalizing, externalizing, and total problems scales. The version for school-aged youths consists of 118 items with syndrome scales (anxious/depressed; withdrawn/depressed; somatic complaints; social problems; thought problems; rule-breaking behavior; attention problems; aggressive behavior), a total problem score, and two broad-band scores (internalizing problems and externalizing problems). The analysis of CBCL scores was performed for the two broad-band scales.

A subsample (63 mothers) completed the Social Communication Questionnaire (SCQ) [80], a 40-item parent-report questionnaire that evaluates three major aspects of ASD: communication, social interaction, and repetitive behaviors. The questionnaire exists in two forms: lifetime and current. The “lifetime” form evaluates the patient’s developmental history, as well as current behaviors, whereas the “current” form assesses the child’s behavior during the past 3 month period only.

### 2.4. Statistical Analyses

Descriptive statistics were used to analyze demographic and clinical characteristics of the whole sample. Pearson and Spearman correlation analyses were used to evaluate the relationship between PSI scores and the identified variables. A Bonferroni correction for multiple comparison was applied, and, according to the number of comparisons, a different *p*-value was considered statistically significant. Analyses of covariance (ANCOVAs) were run with PSI subscales as the dependent variable, employment status (employed vs. non employed)/SES as the fixed factors, and covariates of child individual and clinical features, chosen for each PSI subscale according to the results of the correlation analyses, as well as mother’s age and educational level.

## 3. Results

### 3.1. Maternal Stress

**Figure 1:**
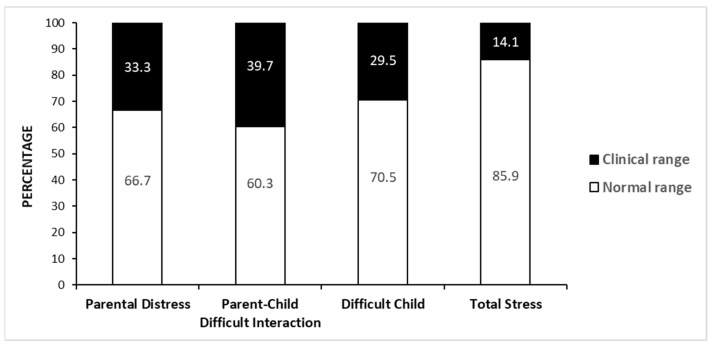
Distribution of the Parenting Stress Index scores (%).

The percentage of mothers who exhibited scores in the clinical range at the PSI scales is reported in Figure 1. The highest prevalence of clinically significant scores was reported for the subscale “Parent–Child Difficult Interaction” (39.7%).

### 3.2. Association between Maternal Stress and Child Features

In order to determine the relationship between maternal stress and selected child individual features (i.e., sex, age, IQ, adaptive level), we performed Spearman and Pearson correlations. Our analysis failed to detect a significant association between PSI scores and sex or IQ; we only observed a weak negative correlation between IQ and the scores obtained at the P-CDI subscale (*r* = −0.228, *p* = 0.049), which did not resist Bonferroni correction. Conversely, significant positive correlations emerged between age and PD (*r* = 0.277, *p* = 0.014), DC (*r* = 0.265, *p* = 0.019), and PSI total stress (*r* = 0.289, *p* = 0.01). However, such correlations did not resist Bonferroni correction. Lastly, we detected significant negative associations between the adaptive abilities, as measured with the General Adaptive Composite score of the ABAS II questionnaire, and all of the PSI subscale scores: PD *r* = −0.259, *p* = 0.023 (did not survive Bonferroni correction); P-CDI *r* = −0.491, *p* < 0.001; DC *r* = −0.326, *p* = 0.004; PSI total stress *r* = −0.359, *p* = 0.001.

### 3.3. Association between Maternal Stress and Child Emotional and Behavior Problems

The presence of associations between maternal stress and child emotional and behavior problems was also investigated. Overall, 33.3% of children in our sample exhibited scores in the clinical range on the internalizing scale of CBCL, whereas 14.1% and 56.6% exhibited scores in the borderline and normal range, respectively. On the other hand, 19.3% of children in our sample displayed scores in the clinical range on the externalizing problems scale of CBCL, whereas 17.9% and 62.8% displayed scores in the borderline and normal range, respectively. As concerns the SCQ questionnaire, 45 out of the 63 patients (71.4%) whose mothers completed the questionnaire obtained scores in the normal range, whereas the remaining 18 (28.6%) exhibited scores in the clinical range; of note, no children in our sample were diagnosed with co-occurring ASD. Results of the correlation analyses are summarized in Table 1. We found that PD was significantly associated only with social communication deficits, whereas the remaining PSI scales were strongly associated with internalizing and externalizing problems.

### 3.4. Association between Maternal Stress and Socio-Demographic Factors

In order to determine the relationship between maternal stress and selected socio-demographic factors (i.e., maternal age and years of education, socioeconomic rank) we performed Spearman and Pearson correlations. We did not detect a significant association between PSI scores and maternal age, mother’s years of education, or socioeconomic rank (all *p* > 0.05).

### 3.5. Differences in PSI Scores between Employed and Unemployed Mothers

In order to investigate the presence of differences in the levels of maternal stress between employed and unemployed mothers of children with DS, four ANCOVAs were run. Considering the statistically significant association that emerged between maternal stress and autistic symptoms, we performed such analyses only in the subgroup of patients for which SCQ data were available.

As concerns the PD scale, covariates of children’s social communication abilities, as well as mother’s age and education levels, were included. We detected a main effect of the employment status, F(1, 57) = 5.61, *p* = 0.021, ηp^2^ = 0.017 (unemployed mothers: 70.43 ± 31.78; employed mothers 54.43 ± 27.39).

As concerns the P-CDI subscale, covariates of children’s adaptive level, internalizing problems, externalizing problems, and mother’s age and education levels were included. There was no main effect of the employment status, F(1, 68) = 0.34, *p* = 0.560, ηp^2^ = 0.003.

As concerns the DC subscale, covariates of children’s adaptive level, internalizing problems, externalizing problems, and mother’s age and education levels were included. There was no main effect of the employment status, F(1, 68) = 0.67, *p* = 0.796, ηp^2^ = 0.038. Lastly, as concerns the total stress subscale, covariates of children’s adaptive level, internalizing problems, externalizing problems, and social communication abilities as well as mother’s age and education levels, were included. There was no main effect of the employment status, F(1, 52) = 2.73, *p* = 0.104, ηp^2^ = 0.070.

### 3.6. Differences in PSI Scores between Mothers with High and Low SES

In order to investigate the presence of differences in the levels of maternal stress between mothers of children with DS belonging to families with high SES and mothers of children with DS belonging to families with low SES, four ANCOVAs were run. Considering the important association emerged between maternal stress and autistic symptoms, we performed such analyses only in the subgroup of patients for which SCQ data were available.

As concerns the PD scale, covariates of children’s age, adaptive level, externalizing problems social communication abilities, and mother’s age and education levels were included. There was no main effect of SES, F(1, 57) = 2.87, *p* = 0.096, ηp^2^ = 0.014.

As concerns the P-CDI subscale, covariates of children’s adaptive level, internalizing problems, externalizing problems, and mother’s age and education levels were included. There was no main effect of SES, F(1, 68) = 0.27, *p* = 0.604, ηp^2^ = 0.004.

As concerns the DC subscale, covariates of children’s adaptive level, internalizing problems, externalizing problems, and mother’s age and education levels were included. There was no main effect of SES, F(1, 68) = 1.90, *p* = 0.172, ηp^2^ = 0.037.

Finally, as concerns the total stress subscale covariates of children’s adaptive level, internalizing problems, externalizing problems, social communication abilities, and mother’s age and education levels were included. There was no main effect of SES, F(1, 52) = 0.05, *p* = 0.832, ηp^2^ = 0.064.

## 4. Discussion

The present study investigated maternal stress levels and their correlates in a group of mothers of youths with DS. We found that parent–child difficult interaction was the PSI domain with the highest percentage of clinical scores (39.7%), followed by the parental distress subscale (33.3%). Significant associations between maternal stress and children’s individual and clinical features emerged; in particular, both internalizing and externalizing problems correlated with maternal stress, as well as ASD symptoms. Conversely, the levels of maternal stress were not associated with any socio-demographic variable. After controlling for child-related correlates of maternal stress and for mothers’ age and education level, we found that unemployed mothers exhibited higher levels of parental distress than employed mothers.

We observed a percentage of mothers exhibiting clinical scores of total stress (14.1%) similar to those previously reported in the literature on DS [48]. This seems to support the idea of a *Down Syndrome advantage* [44]. However, looking at the single PSI subscales, our results indicated a higher percentage of mothers exhibiting clinical scores in the P-CDI subscale; this suggests that nearly 40% of mothers in our group of participants showed some feelings of disappointment or rejection to the child, as indicated by the subscale [2]. This finding is consistent with previous research suggesting that the effects of the *Down Syndrome advantage* could be diminished or rooted out if appropriate controls between etiology groups are employed. For instance, Cahill and Glidden [81] compared groups of parents of children with DS with two mixed-etiology groups of children with developmental disabilities and found that the *Down Syndrome advantage* disappeared after matching for child level of functioning, child’s age, parental marital status, and family income. These results were replicated in subsequent studies [72,82]. A more recent study comparing mothers of children with DS with mothers of children with ID of mixed etiologies reported that the initial group differences in psychological distress and life satisfaction were largely associated with family poverty [83]. In summary, it emerges that research on parenting stress levels in DS needs to take into account SES when comparing reports from parents of children with DS with parents of children with other genetic conditions and/or other IDs.

Of note, the literature indicates that SES affects parenting stress in NDDs [65,66,68]. In the present study, no association between the levels of maternal stress and SES or maternal education emerged. Thus, it is supposable that SES is an important variable to consider when comparing parenting stress levels across syndromes but it does not have a crucial role *per se* in modulating maternal stress levels in DS. This is further supported by the ANCOVAs performed in the present study, which did not reveal any difference in maternal stress levels between mothers of children with SD belonging to families with high SES compared with families with mothers belonging to families low SES.

Children’s individual and clinical features also play a key role in modulating parenting stress. The levels of functional impairment and behavioral problems, indeed, are other factors commonly reported in association with parenting stress in NDDs such as ASD [84] and ADHD [85], but not developmental coordination disorder [86]. In the current study, children’s individual factors most consistently associated with the various facets of parenting stress were the adaptive abilities. The association between maternal stress and children’s adaptive abilities is not surprising, considering that higher levels of functional impairment usually result into higher demands to parents. This is particularly true for individuals with DS, given the high rates of medical comorbidities [87]. Our results did not show an association between children’s sex and the levels of parenting stress, consistently with previous research [26]. However, this could be due to the higher proportion of males included in our sample; future research should point to involving a larger sample with equal sex distribution.

As concerns the association between maternal stress and children’s behavioral problems, our findings linking maternal stress to children’s externalizing symptoms are consistent with a body of literature that indicates a correlation between externalizing symptoms and parental stress levels both in TD [88] and in a wide range of NDDs and genetic diseases, such as ASD [88,89,90], ADHD [24,85,91], language disorder [24], Angelman syndrome [92], and WS [48,93]. This provides clear indications for future studies for taking into account the measurement of parental stress when evaluating the effects of interventions focused on externalizing symptoms.

In addition to the current literature, this study supports the contribution of internalizing problems of youths with DS for parental stress. The literature on NDDs indicates that externalizing behaviors seem to be more strongly associated with parenting stress than internalizing symptoms [89]. However, in the DS population, research on the association between parental stress and internalizing symptoms has provided inconsistent results. Our study offers new insights into the effect of children’s internalizing problems on maternal stress in youths with DS. Taking into account the well-documented transition toward internalizing problems for adolescents with DS, this finding must be considered carefully [94,95].

A significant correlation between social communication difficulties, as measured through the SCQ, and scores obtained at the PD scale emerged. This finding is consistent with previous research suggesting that the presence of ASD symptoms in children is associated with increased parental stress levels in the ID population [96]. However, to the best of our knowledge, this is the first study assessing the levels of parenting stress in relation to ASD symptoms in mothers of youths with DS without comorbid ASD. This result underlines the importance of considering social communication difficulties in intervention programs for DS families, even in absence of comorbid ASD.

Concerning the literature indicating an impact of NDDs on parents’ employment, we also asked if there was any difference in the levels of parenting stress between employed and unemployed mothers. After controlling for children’s features associated with maternal stress and maternal age and educational level, results documented a significant difference in parenting distress between employed and unemployed mothers. Conversely, we observed that SES is a situational life/demographic factor not related with maternal stress in DS, consistently with some previous findings [38]. This finding is especially relevant considering that parents of individuals with NDDs are often at increased risk of losing their job, cutting down on hours worked, or changing jobs [97,98,99,100,101]. In particular, mothers having a lifelong care responsibility for children with NDDs are at a greater risk of unemployment or part-time employment than mothers of children without NDDs [102,103,104,105,106]. Job loss has been negatively related with mental health outcomes, in terms of higher depressive symptoms, anxiety, psychosomatic symptoms, subjective wellbeing, and self-esteem [107,108,109]. Intriguingly, we found that employed mothers and unemployed mothers only differed in the PD scale of the PSI, which captures the level of distress linked with conflicts with the partner, social support, and distress caused by life restrictions due to child rearing; thus, such a scale is relatively independent from children’s features [71]. Our results could be explained, at least in part, by the low satisfaction linked to the unemployment condition *per se*, especially for cases in which the condition was subsequent to the birth of the child with DS. This interpretation grounds on previous research indicating that parental unemployment is associated with higher levels of parenting stress [110], and that employed parents, especially mothers, often exhibit lower stress than unemployed parents [111]. Another aspect to take into account is related to the conceivable higher burden required by unemployed mothers for long-term continuous care for their child. For instance, the higher rates of medical comorbidities in children with DS often requires parents to spend a huge amount of time for medical examinations and, in many cases, also to travel and stay away from their homes while receiving care for their children. Therefore, it is supposable that parenting a child with DS could become an overwhelming experience for unemployed mothers. The results of this study also lead to some considerations about the role of gender issues. The higher levels of parental distress observed in unemployed mothers could also be interpreted in the light of some gender differences occurring in children care. The gender issue in children care has been little addressed in the SD literature. The little evidence available emphasizes that mothers of children with a form of disability, including DS, are more involved than fathers in their child’s intervention and childrearing practices [27,112,113]. It is possible to hypothesize that mothers most often provide general physical care of the child while at home, with consequent increased burden. However, this issue needs to be further and more thoroughly explored, and research comparing maternal and paternal stress and their respective involvement in child care is needed.

Before discussing the implications of the current study, we comment on some important limitations. Firstly, no control group was included for comparison. Since many neurogenetic syndromes are associated with specific cognitive and behavioral phenotypes [32], comparative research examining possible sources of stress in caregivers of children with different disorders is needed. Secondly, behavioral and emotional problems were assessed using only parental reports. It is crucial to validate our findings with methods that consider multiple independent respondents, such as reports from teachers, as well as to investigate methodologies other than questionnaires such as direct home- or school-based observation to obtain a better understanding of the frequency, severity, and duration of these challenging behaviors. Although clinical evaluations excluded significant psychopathological comorbidity in our sample, we investigated the presence of an association between parental perception of child’s behavioral symptoms and maternal stress without focusing on a possible causal link, for which the use of multiple independent respondents would have been crucial, especially for a cross-sectional design. Thirdly, although we did not detect significant associations between children’s age and the levels of maternal stress, the large age range of the participants included could be a weakness of this study. Indeed, the factors associated with maternal stress may vary with the behavioral changes occurring with age in DS population (e.g., the switch toward internalizing behaviors) and possible situational life/demographic modifications interacting with them. Therefore, further and, possibly, longitudinal studies are required to explore age-related variables linked with parenting stress in mothers of youths with DS. Lastly, we found a relatively high percentage of children with SCQ scores in the pathological range. Of note, children with a formal comorbid diagnosis of ASD were excluded from the study; moreover, the percentage of children exhibiting high ASD symptoms is not dissimilar from that observed in previous studies using parent-report questionnaires to detect ASD symptoms in youths with DS without comorbid ASD [114,115].

Nevertheless, to the best of our knowledge, this is the first study highlighting differences in parenting stress related to the situational life/demographics condition of employment in mothers of children with DS. Implications of our findings can be applied to clinical practice and health services concerning care–work reconciliation for mothers with lifelong caregiving responsibilities. The present study highlights that unemployment is related to parenting stress and potentially amenable to policy interventions supporting parents in combining work and family care, for instance, by providing families with affordable daycare. Indeed, policy interventions should consider that parents’ wellbeing has repercussions not only for the parents themselves but also for child development. On the other hand, our results also provide indications for families of children with DS; in particular, parents should complete an accurate reflection about the decision to leave the job in favor of devoting all their time to child care.

Future research should focus on psychological variables (e.g., coping strategies) possibly mediating the differences in parenting stress between employed and unemployed mothers of children with DS. Moreover, additional studies are required to investigate paternal stress in children and adolescents with DS and to explore possible differences in both levels and correlates in comparison with maternal stress.

## 5. Conclusions

The results of the present study are consistent with the large body of evidence that indicates how externalizing problems are associated with higher levels of maternal stress in DS; they also provide additional evidence to the current literature for an effect of internalizing problems and social communication deficits on maternal stress levels, highlighting the importance of proper interventions for these aspects, as well as for the improvement of family wellbeing in DS. Moreover, unemployed mothers exhibited significantly higher levels of stress than employed mothers linked to personal factors, such as life restrictions due to the requests of child rearing. This finding provides useful indication for policy interventions aimed at improving the wellbeing of families of youths with DS. Further work is necessary to explore the levels of parenting stress in fathers, age-specific factors, and psychological variables (coping strategies) associated with parenting stress in DS. Considerations for clinical practice and health services emerge from our findings, suggesting that assessing parental stress levels in clinical practice is informative to define appropriate treatment and support plans for youths with DS and their families.

## Figures and Tables

**Table 1:** Association between maternal stress and child emotional and behavioral problems.

	Parental Distress *r* (*p*)	Parent–Child Difficult Interaction *r* (*p*)	Difficult Child *r* (*p*)	Total Stress *r* (*p*)
Internalizing problems (CBCL)	0.281 * (0.013)	0.363 ** (0.001)	0.465 ** (<0.001)	0.415 ** (<0.001)
Externalizing Problems (CBCL)	0.313 * (0.005)	0.394 ** (<0.001)	0.623 ** (<0.001)	0.550 ** (<0.001)
SCQ	0.399 ** (<0.001)	0.338 * (0.007)	0.336 * (0.007)	0.375 ** (0.002)

* *p* < 0.05, ** survived Bonferroni correction (*p* ≤ 0.004). CBCL: Child Behavior Checklist; SCQ: Social Communication Questionnaire.

## Data Availability

The data presented in this study are available on request from the corresponding author.
